# Beyond expansion: the strategic role of the tissue expander in immediate breast reconstruction

**DOI:** 10.1016/j.jpra.2026.09.027

**Published:** 2026-09-23

**Authors:** Franck Dupuy, Guillaume Henry, Thomas Dinahet, Mathilde Lherm, Fabien Boucher, Haïzam Oubari, Ali Mojallal, Hugo Despert

**Affiliations:** a Hospices civils de Lyon, Plastic Aesthetic and Reconstructrive Surgery, Croix Rousse Hospital, 103 Grande rue de la Croix-Rousse, 69000, Lyon, France; b Université Claude Bernard Lyon 1, 69100, Villeurbanne, France

**Keywords:** Immediate breast reconstruction, Tissue expander, Postmastectomy radiotherapy, Direct-to-implant reconstruction, Delayed-immediate breast reconstruction

## Abstract

Background: Immediate tissue expander breast reconstruction remains widely used after mastectomy, but its contemporary role extends beyond progressive skin-envelope expansion. By providing adjustable volume and tension control, accommodating oncological sequencing, and preserving future reconstructive options, the tissue expander may serve as a strategic tool when the definitive reconstructive pathway remains uncertain. This focused narrative review evaluates the factors guiding immediate reconstructive strategy, integration with postmastectomy radiotherapy (PMRT), comparison with direct-to-implant (DTI) reconstruction, and the role of tissue expanders in delayed-immediate reconstruction. Methods: A structured literature search of PubMed/MEDLINE and Embase identified publications from January 2010 to June 2026. Systematic reviews, meta-analyses, comparative cohorts, multicentre studies, and studies reporting patient-reported outcomes were prioritised and synthesised thematically around four predefined clinical questions. Results: No reconstructive strategy demonstrates universal superiority. DTI provides an efficient definitive option when breast anatomy, tissue conditions, patient risk profile, and the oncological pathway are favourable and predictable. Tissue expander reconstruction offers greater adjustability when these conditions are less favourable or remain uncertain. In patients receiving PMRT, irradiation of the expander is associated with a higher risk of reconstructive failure, whereas irradiation of the permanent implant is associated with more severe capsular contracture; however, prospective multicentre studies have not consistently demonstrated differences in failure after adjustment for confounding factors. Delayed-immediate reconstruction preserves the breast envelope and maintains both implant-based and autologous options while definitive treatment is clarified, although the additional expander stage introduces specific morbidity and resource utilisation. Conclusions: Tissue expander reconstruction should not be regarded as a default intermediate step or a universally safer alternative to definitive reconstruction. Its contemporary value lies in preserving reconstructive flexibility when anatomical, oncological, or patient-related factors make immediate commitment to a definitive strategy less appropriate. Future studies should identify the patients in whom this adjustability translates into meaningful clinical benefit.

## Introduction

Immediate breast reconstruction has become a central component of the comprehensive management of patients undergoing mastectomy for breast cancer. When oncologically appropriate and technically feasible, it enables early restoration of breast volume and contour while mitigating the psychological and body-image consequences of mastectomy. Immediate reconstruction has therefore been associated with improvements in body image, patient satisfaction, and several dimensions of quality of life.^1^^,^^2^ Over the past decades, reconstructive techniques have evolved considerably, providing a broad range of autologous, implant-based, and hybrid options.

Within implant-based reconstruction, two-stage reconstruction with initial placement of a tissue expander remains a widely used strategy.^3^^,^^4^ Although direct-to-implant (DTI) reconstruction has gained increasing popularity, particularly with the development of acellular dermal matrices and prepectoral techniques, tissue expander reconstruction continues to retain a distinct role in immediate breast reconstruction.^5^ Its principal advantage is its adjustability: breast volume and soft-tissue tension can be progressively modified according to mastectomy-flap quality, patient anatomy, and the anticipated oncological treatment pathway.^3^, ^4^, ^5^

Importantly, the contemporary role of the tissue expander extends beyond progressive expansion of the mastectomy skin envelope. By providing temporary, adjustable volume control, it can reduce mechanical stress on compromised mastectomy flaps, preserve the breast envelope, and defer the definitive reconstructive strategy until local and oncological conditions are better defined. This flexibility may be particularly valuable when tissue viability is uncertain, when the indication for postmastectomy radiotherapy (PMRT) remains unresolved, or when the definitive choice between implant-based and autologous reconstruction has not yet been established.^5^^,^^6^ The tissue expander should therefore be considered not only as an expansion device, but also as a strategic means of managing reconstructive uncertainty.

Despite this evolving role, recent reviews have generally addressed implant-based breast reconstruction as a whole.^7^^,^^8^ or focused on specific technical questions, such as the prepectoral–subpectoral approach,^9^^,^^10^ rather than on the tissue expander itself. Its contemporary indications and strategic applications in immediate breast reconstruction therefore remain incompletely defined. This narrative review addresses four questions: Which factors should guide the choice of immediate reconstructive strategy after mastectomy? How should tissue expander reconstruction be integrated with postmastectomy radiotherapy? How does two-stage reconstruction compare with direct-to-implant reconstruction ? Finally, what evidence supports the use of the tissue expander as a bridging device in delayed-immediate reconstruction?

## Methods

This focused narrative review addressed four predefined clinical questions: factors guiding immediate reconstructive strategy after mastectomy, integration of tissue expander reconstruction with postmastectomy radiotherapy, comparison with direct-to-implant reconstruction, and the role of the tissue expander in delayed-immediate reconstruction. Recent technical developments were also considered.

PubMed/MEDLINE and Embase were searched for articles published between January 2010 and June 2026, supplemented by earlier seminal studies. Core search terms included “breast reconstruction”, “immediate breast reconstruction”, “tissue expander”, and “expander-based reconstruction”, combined with topic-specific terms related to patient selection, radiotherapy, direct-to-implant reconstruction, and delayed-immediate reconstruction. Reference lists of relevant reviews and key articles were also screened.

Relevant evidence included systematic reviews, meta-analyses, prospective and retrospective comparative studies, multicentre cohorts, and studies reporting patient-reported outcomes. Preference was given to larger, recent, methodologically robust studies with adjusted analyses, longer follow-up, or validated outcome measures.

The aim was to identify the most clinically relevant evidence rather than to perform an exhaustive systematic review. Studies were grouped by topic, with particular attention to study design, follow-up, and possible sources of bias or heterogeneity.

## Results

### Patient, breast, and treatment related factors guiding immediate breast reconstruction

Immediate breast reconstruction was historically limited by oncological and technical concerns, particularly the risk that postoperative complications might delay adjuvant treatment and that PMRT could compromise reconstructive outcomes. These issues remain relevant, especially as the indication for PMRT may only become apparent after definitive pathological assessment.^6^^,^^11^

Nevertheless, accumulating evidence has established immediate breast reconstruction as an integral component of therapeutic and risk-reducing mastectomy pathways, without adverse effects on survival, locoregional recurrence, or oncological surveillance.^1^^,^^12^^,^^13^ By preserving the breast envelope and combining oncological and reconstructive surgery, it may also reduce the overall reconstructive burden and improve body image, satisfaction, and quality of life.^1^^,^^2^^,^^11^

Beyond this oncological reassurance, technical advances have improved the safety, predictability, and aesthetic outcomes of immediate breast reconstruction. Skin-sparing mastectomy and, in appropriately selected patients, nipple-sparing mastectomy preserve the breast skin envelope, inframammary fold, and nipple–areola complex, thereby improving breast shape, projection, and aesthetic outcomes.^12^^,^^14^^,^^15^ In parallel, advances in implant technology, acellular dermal matrices and synthetic meshes, fat grafting, and prepectoral reconstruction have further improved the predictability and aesthetic outcomes of implant-based breast reconstruction.^16^^,^^17^ Prepectoral placement avoids disruption of the pectoralis major muscle and may reduce postoperative pain, animation deformity, and functional morbidity, while facilitating a more natural breast contour in appropriately selected patients.^18^

These developments have extended immediate reconstruction beyond the traditional profile of patients with small, non-ptotic breasts.^19^ However, reconstructive planning must remain individualized, taking into account breast morphology, mastectomy-flap quality, patient-related risk factors, and the anticipated oncological treatment pathway. Unfavourable anatomy, impaired tissue healing, and exposure to radiotherapy may each increase the risk of wound complications, necrosis, implant exposure, and reconstructive failure. Reported rates place these risks in perspective: in a systematic review of 59 studies and 14,368 patients, mastectomy flap necrosis occurred in 10.4% of breasts (range 2.3–41.2%), the width of that range itself reflecting the absence of a standardised definition.^20^

These factors have direct implications for reconstructive planning. In patients undergoing therapeutic mastectomy, large breast volume, marked ptosis, uncertain mastectomy-flap viability, or a high risk of postoperative complications may favour a staged approach, including skin-reducing mastectomy or two-stage expander-based reconstruction, in order to reduce mechanical stress on the skin envelope and preserve reconstructive flexibility. Likewise, when radiotherapy is anticipated or its indication remains uncertain, the reconstructive sequence should be discussed preoperatively within a multidisciplinary team.^5^^,^^18^

Closer collaboration between breast surgeons, reconstructive surgeons, medical oncologists, and radiation oncologists has facilitated the integration of immediate reconstruction into the overall treatment pathway. However, access remains uneven, and substantial disparities persist between healthcare institutions.^21^ Immediate breast reconstruction should therefore be discussed with every patient undergoing mastectomy.^6^^,^^18^

In practice, three domains carry particular weight when selecting the immediate reconstructive strategy: the anticipated need for PMRT, the patient’s breast anatomy and comorbidity profile, and whether the definitive reconstructive strategy has already been established. These factors determine whether definitive reconstruction can reasonably be undertaken at mastectomy or whether a staged, adjustable approach may provide greater flexibility.

### Tissue expander reconstruction and postmastectomy radiotherapy

PMRT is an essential component of treatment for patients at increased risk of locoregional recurrence and may improve both disease control and survival.^22^^,^^23^ Its indication may only become apparent after definitive pathological assessment. This uncertainty is particularly relevant in immediate breast reconstruction because PMRT impairs tissue healing and promotes fibrosis, increasing wound complications, capsular contracture, revision surgery, and reconstructive failure. It is also associated with poorer aesthetic outcomes and patient-reported satisfaction.^22^, ^23^, ^24^, ^25^

In two-stage expander–implant reconstruction, PMRT may be delivered to the tissue expander before exchange or to the permanent implant after exchange.^23^^,^^26^ Irradiating the expander avoids an additional operation before PMRT and allows capsulotomy or capsulectomy at subsequent exchange**.** Conversely, irradiating the permanent implant avoids operating on recently irradiated tissues but exposes the definitive reconstruction to radiation-induced fibrosis**.** The principal comparative studies evaluating these sequences are summarised in Table 1.

**Table 1:** Postmastectomy radiotherapy sequencing: expander versus permanent implant.

First author	Year	Journal	Study design	N (irradiated)	Plane	Timing of PMRT	Overall complications	Reconstructive failure	Severe capsular contracture	PROMs	LoE
Nava	2011	Plastic Reconstr Surg	Single-centre cohort	50 TE-XRT; 109 PI-XRT 98 no XRT	Subpectoral	TE-XRT vs PI-XRT	NR	**40.0% (20/50) TE-XRT vs 6.4% PI-XRT** (**2.3% no XRT); p<0.0001**	**13.3% TE-XRT vs 10.1% PI-XRT p<0.01**	Patient’s opinion of final result rated as good 52.2% PI-XRT vs 46.2% TE-XRT	III
Cordeiro	2015	Plastic Reconstr Surg	Single-surgeon retrospective cohort (2003–2012)	94 TE-XRT; 210 PI-XRT 1486 no XRT	Subpectoral	TE-XRT vs PI-XRT	NR	**18.1% TE-XRT vs 12.4% PI-XRT; No significant difference**	**1.22% TE-XRT vs 6.3% PI-XRT p<0.01**	BREAST-Q comparable between sequences. Aesthetics very good–excellent 75.0% TE-XRT vs 67.6% PI-XRT; p<0.01	III
Santosa	2016	Plastic Reconstr Surg	MROC prospective multicentre cohort (11 sites)	104 TE-XRT; 46 PI-XRT	NR	TE-XRT vs PI-XRT	**No significant difference**	**11.5% TE-XRT vs 8.7% PI-RXT No significant difference**	**2.9% TE-XRT vs 2.2% PI-XRT**	NR	II
Ricci	2017	Journal Surg Research	Systematic review + meta-analysis	1479 TE-XRT; 869 PI-XRT	NR	TE-XRT vs PI-XRT	NR	**20.0% TE-XRT vs 13.4% PI-XRT; p=0.001**	**24.5% TE-XRT vs 49.4% PI-XRT**	NR	II
Yoon	2020	Plastic Reconstr Surg	MROC prospective multicentre cohort (11 sites)	237 TE-XRT; 80 PI-XRT	NR	TE-XRT vs PI-XRT	**No significant difference**	**19.8% TE-XRT vs 10% No significant difference after controlling covariates**	**NR**	BREAST-Q, PROMIS, EORTC QLQ-BR23: no significant difference in most domains. Satisfaction with breasts and physical/sexual well-being declined after PMRT in both groups	II
Ogita	2025	Breast Cancer	Systematic review + meta-analysis (JBCS guideline)	623 TE-XRT; 670 PI-XRT for reconstruction failure analysis	NR	TE-XRT vs PI-XRT	**No significant difference**	**19.9% TE-XRT vs 11.8% PI-XRT; p=0.0007**	**23.4% TE-XRT vs 52.7% PI-XRT**	No significant difference	II

Comparative studies of postmastectomy radiotherapy sequencing in two-stage implant-based breast reconstruction. Studies are ordered by year of publication. Two distinct sequences are compared throughout: delivery of radiotherapy to the tissue expander before exchange (TE-XRT) and delivery of radiotherapy to the permanent implant after exchange (PI-XRT).

*Abbreviations:* BREAST-Q, breast reconstruction patient-reported outcome measure; EORTC QLQ-BR23, European Organisation for Research and Treatment of Cancer Quality of Life Questionnaire Breast Cancer Module; JBCS, Japanese Breast Cancer Society; LoE, level of evidence; MROC, Mastectomy Reconstruction Outcomes Consortium; NR, not reported; No XRT, No radiotherapy; PI-XRT, radiotherapy delivered to the permanent implant; PMRT, postmastectomy radiotherapy; PROMIS, Patient-Reported Outcomes Measurement Information System; PROMs, patient-reported outcome measures; TE-XRT, radiotherapy delivered to the tissue expander.

Earlier single-centre studies suggested a trade-off between these strategies. Nava et al. reported a substantially higher reconstructive failure rate when PMRT was delivered to the tissue expander rather than to the permanent implant (40.0% [20/50] vs. 6.4% [7/109]; *p* < 0.0001), compared with 2.3% in a non-irradiated control group.^27^ However, the non-randomised cohorts represented different oncological settings, with locally advanced disease and more urgent PMRT in the tissue-expander group. Similarly, Cordeiro et al.^28^ reported a higher predicted 6-year failure rate after tissue-expander irradiation (32.0% vs. 16.4%; *p <* 0.01), whereas severe capsular contracture was more frequent after permanent-implant irradiation (Baker III: 44.6% vs. 15.9%; Baker IV: 6.3% vs. 1.2%; *p <* 0.01). These studies therefore associated tissue-expander irradiation with greater reconstructive failure and permanent-implant irradiation with more severe capsular contracture, although both reflected older subpectoral techniques and non-randomised treatment sequences.

More recent prospective multicentre evidence has provided a more nuanced picture. In the Mastectomy Reconstruction Outcomes Consortium, Santosa et al.^26^ found no significant difference in reconstructive failure between irradiation at the tissue-expander and permanent-implant stages (11.5% vs. 8.7%; *p* = 0.900), including after adjustment. In a subsequent analysis of 317 patients, Yoon et al.^29^ observed a higher unadjusted failure rate after tissue-expander irradiation (19.8% vs. 10.0%; *p =* 0.04), but this association disappeared after multivariable adjustment (OR 0.72, 95% CI 0.28–1.83; *p =* 0.49). Radiotherapy timing also had little effect on most patient-reported outcomes at two years.

Meta-analytic evidence has nevertheless reproduced the trade-off reported in earlier studies. Ricci et al.^30^ found a higher risk of reconstructive failure after tissue-expander irradiation (20.0% vs. 13.4%; RR 2.33, 95% CI 1.24–4.35; *p =* 0.0083), whereas severe capsular contracture was less frequent (24.5% vs. 49.4%), although this difference did not reach statistical significance (RR 0.53, 95% CI 0.25–1.09; *p =* 0.083). More recently, Ogita et al.^31^ similarly reported higher failure after tissue-expander irradiation (19.9% vs. 11.8%; OR 2.33, 95% CI 1.43–3.82; *p =* 0.0007) but less severe capsular contracture (23.4% vs. 52.7%; OR 0.33, 95% CI 0.12–0.92; *p =* 0.03), with comparable major complication rates. Thus, meta-analyses support the trade-off reported in earlier studies, whereas prospective multicentre analyses have not consistently shown differences in failure after adjustment. No sequence can therefore be considered universally superior.

Anticipated PMRT should be considered when selecting the initial reconstructive strategy, not only when deciding which device should be irradiated. Sequencing should be individualised according to oncological timing, flap quality, comorbidities, reconstructive objectives, and patient preferences. Expansion should be coordinated with radiation oncology, and PMRT should not be delayed solely to permit implant exchange.

When the tissue expander is irradiated, definitive implant exchange is generally postponed until acute radiation effects have subsided. Peled et al.^32^ reported a lower failure rate when exchange was performed at least 6 months after PMRT compared with earlier exchange (7.7% vs. 22.4%; *p =* 0.036), with the highest failure rate when exchange occurred within 3 months (28.6%). A systematic review by Chang et al.^33^ similarly found higher rates of wound dehiscence (17.12% vs. 3.64%; *p <* 0.001) and haematoma (25.0% vs. 2.59%; *p <* 0.001) when exchange occurred within 6 months, without significant differences in infection, seroma, capsular contracture, or reconstructive failure. These findings support an interval of approximately 6 months when circumstances permit, although this should not be considered an absolute threshold. The expander may subsequently be exchanged for either a permanent implant or autologous tissue according to reconstructive objectives, tissue conditions, donor-site availability, and patient preference.^28^^,^^33^, ^34^, ^35^

### Direct-to-implant versus two-stage expander-implant reconstruction

Comparative studies of DTI and two-stage expander–implant reconstruction have generally reported similar overall outcomes, although interpretation is limited by heterogeneous outcome definitions, differences in follow-up, and substantial selection bias. Early retrospective studies suggested a modest increase in early complications and implant loss after DTI reconstruction, without consistent differences in infection or short-term reoperation rates.^36^, ^37^, ^38^ The principal advantages of DTI reconstruction are avoidance of a planned implant-exchange procedure and expansion-related outpatient visits, resulting in a shorter pathway to definitive reconstruction.^39^^,^^40^ Secondary revision procedures may nevertheless still be required.

More recent evidence has highlighted the importance of patient selection. In the prospective cohort study by Chiang et al.,^41^ patients undergoing DTI reconstruction were younger, had fewer comorbidities, and generally had smaller breasts, and were more likely to undergo nipple-sparing mastectomy and prepectoral reconstruction. Similarly, Finkelstein et al.^42^ in a retrospective comparison of 536 immediate prepectoral reconstructions, reported broadly comparable overall outcomes, although postoperative wound complications were significantly more frequent after DTI reconstruction (*p <* 0.0001). Within the DTI group, greater mastectomy resection weight was strongly associated with wound-related complications, supporting the importance of breast volume and tissue burden in patient selection. Riggio et al.^43^ also reported comparable overall complication rates, while identifying higher body mass index, radiotherapy, and device size as risk factors for major complications within the tissue expander group. Collectively, these findings highlight the extent to which patient selection and confounding by indication influence comparisons between the two strategies. These selection factors are synthesised in Table 2, which summarises the clinical characteristics that may favour DTI, tissue expander, or autologous reconstruction.

**Table 2:** Patient and treatment related factors guiding the choice between direct-to-implant, tissue expander, and autologous reconstruction.

Factor	Favours DTI	Favours tissue expander	Favours autologous
**Breast volume**	Small to moderate; limited volume replacement required	Larger volume, where staged control of the skin envelope is needed	Moderate to large volume with adequate donor tissue, allowing volume match without a prosthesis
**Degree of ptosis**	Absent or grade I	Grade I–II, or Grade III if combined with skin-reducing mastectomy	Grade I–III, particularly where a natural ptotic contour is the goal
**Mastectomy flap thickness and perfusion**	Thick, uniformly perfused flaps with no intraoperative concern	Well-perfused mastectomy flaps; if perfusion is uncertain, a low initial fill can be used to limit tension	Vascularised autologous tissue helps secure the reconstruction and allows secondary-intention healing or skin grafting if necessary.
**Mastectomy specimen weight**	Lower resection weight	Higher resection weight	All resection weight with matching donor volume
**Body mass index**	Lower BMI	Elevated BMI, where staged reconstruction limits early complication burden	Mid-range BMI; very low BMI limits donor-site volume, elevated BMI increases microsurgical risk
**Prior radiotherapy**	Not favoured	Not favoured	Vascularised tissue addresses an irradiated, fibrotic envelope
**Anticipated PMRT: indicated**	Accepting an elevated risk of capsular contracture	Acceptable, with sequencing of radiotherapy and exchange planned in advance	Preferred where the patient is a candidate
**Anticipated PMRT: uncertain**	Acceptable, with counselling that staged reconstruction may be required.	Acceptable	Preferred if the patient has already chosen autologous reconstruction irrespective of radiotherapy
**Anticipated PMRT: not indicated**	Attractive option where local factors otherwise allow	When the definitive reconstructive strategy remains undecided	Where the patient prefers a prosthesis-free reconstruction
**Definitive strategy: undecided**	Not appropriate; commits the patient prematurely	Preferred; preserves both implant-based and autologous pathways	Not appropriate until the decision is made
**Definitive strategy: implant decided**	Preferred if anatomy and perfusion allow	Where staged volume and tension control is required	—
**Definitive strategy: autologous decided**	Not appropriate	As a bridging device when reconstruction must be deferred	Performed immediately where oncological and logistic conditions allow
**Donor site availability**	Not applicable	Not applicable	Requires adequate abdominal, thoracodorsal or gluteal donor tissue
**Patient preference and tolerance of staged care**	Preference for the shortest pathway and a single operation	Acceptance of expansion visits and a planned second procedure	Acceptance of a longer operation, a donor site and microsurgical risk
**Institutional organisation**	Requires immediate availability of implant sizing and reconstructive expertise	Lowest organisational threshold; expansion can be completed at a later date and in a different setting	Requires microsurgical capability, theatre time and postoperative monitoring

Patient, tumour and treatment related factors guiding the choice between direct-to-implant, two-stage tissue expander and autologous reconstruction at the time of mastectomy. This table synthesises the direction in which each factor pushes the decision; Three factors carry disproportionate weight in practice and should be assessed in sequence: the certainty of the postmastectomy radiotherapy indication, the patient’s breast anatomy and soft-tissue characteristics, and whether the definitive reconstructive strategy has been agreed with the patient.

*Abbreviations:* BMI, body mass index; DTI, direct-to-implant; PMRT, postmastectomy radiotherapy.

Prospective multicentre data from the Mastectomy Reconstruction Outcomes Consortium further support broadly comparable outcomes. Srinivasa et al.^44^ compared 99 DTI with 1,328 two-stage expander–implant reconstructions and found no significant differences in overall complications, major complications, infection, or reconstructive failure at two years after multivariable adjustment. Patient-reported outcomes were also comparable across most BREAST-Q domains. According to the minimal important differences (MID) subsequently established by Voineskos et al.^45^, 4 points for Satisfaction with Breasts, Psychosocial Well-Being, and Sexual Well-Being, and 3 points for Physical Well-Bein, the adjusted differences in breast satisfaction (+3.46), psychosocial well-being (+0.85), and physical well-being (+1.29) remained below clinically meaningful thresholds. Only sexual well-being favoured DTI reconstruction by 5 points (*p =* 0.047), slightly exceeding the estimated MID for this domain. Thus, most observed differences in patient-reported outcomes are unlikely to represent clinically meaningful advantages of one strategy over the other.

At a broader level, a systematic review and meta-analysis of 33 comparative studies including 21,529 patients found no evidence of overall superiority of either one-stage or two-stage implant-based reconstruction.^46^ Pooled analyses showed no significant differences in overall complications (29–34%; *p =* 0.80) or implant loss (5–10%; p= 0.66), while patient-reported satisfaction and aesthetic outcomes were likewise comparable. However, substantial heterogeneity and the predominance of subpectoral reconstructions limit extrapolation to contemporary prepectoral practice.

Taken together, these data support a selective rather than competitive view of DTI and tissue expander reconstruction. DTI reconstruction represents an efficient definitive option when breast anatomy, mastectomy-flap perfusion, patient risk profile, and the oncological pathway are favourable and predictable. Conversely, tissue expander reconstruction provides an adaptive strategy when these conditions are less favourable or remain uncertain, allowing breast volume and soft-tissue tension to be adjusted while preserving subsequent reconstructive options. Rather than representing an inferior substitute for DTI reconstruction, the tissue expander should therefore be regarded as a deliberate strategy for managing reconstructive uncertainty.

## Delayed-immediate breast reconstruction

The delayed-immediate strategy aims to preserve the advantages of reconstruction initiated at the time of mastectomy while deferring definitive reconstruction until the oncological pathway is clarified. It involves temporary placement of a tissue expander to maintain the breast skin envelope and reconstructive space pending final pathology and adjuvant treatment planning.^47^, ^48^, ^49^, ^50^, ^51^, ^52^ In the original protocol, the tissue expander was conceived not primarily as a device for progressive expansion, but as an adjustable scaffold preserving the three-dimensional skin envelope and key anatomical landmarks until the need for PMRT was established.^48^ If radiotherapy was not required, definitive reconstruction could proceed early; when PMRT was indicated, the expander served as a temporary bridge before delayed autologous reconstruction.

This strategy is particularly relevant when the indication for PMRT remains uncertain at the time of mastectomy. If radiotherapy is subsequently required, it can be delivered to the tissue expander rather than to a definitive autologous reconstruction, allowing microsurgical reconstruction to be deferred until completion of oncological treatment and reassessment of local tissue conditions.^49^^,^^50^ Maintaining the breast envelope during this interval may limit skin contraction and reduce the amount of skin that must subsequently be replaced. The expander therefore acts as an adjustable bridging device, preserving reconstructive volume and space while allowing donor sites, patient preferences, and the definitive reconstructive strategy to be reassessed.^48^, ^49^, ^50^, ^51^^,^^53^ Contemporary experience also illustrates that this strategy does not necessarily commit the patient to autologous reconstruction: in a series of 115 patients undergoing delayed-immediate reconstruction with a temporary subcutaneous implant, 76 completed definitive reconstruction, including 26 with autologous tissue, 21 with a latissimus dorsi flap and implant, and 28 with an implant alone.^54^

The morbidity associated with this additional reconstructive stage must, however, be acknowledged. In a retrospective comparison of staged tissue-expander/free-flap and purely delayed free-flap reconstruction, Shammas et al.^55^ found higher rates of surgical-site infection (40.7% vs. 6.5%), wound-healing complications (29.6% vs. 12.3%), and return to the operating room (27.8% vs. 4.4%) with the staged approach; most complications occurred during the expansion phase, which was associated with an 18.5% tissue-expander failure rate. Importantly, once patients reached definitive flap reconstruction, microsurgical complications, flap loss, and fat necrosis were comparable between groups.^55^ In a subsequent claims-based analysis of 3,310 patients, staged reconstruction was likewise associated with more surgical complications than immediate free-flap reconstruction (57.8% vs. 32.0%; adjusted RR 1.67, 95% CI 1.49–1.87), as well as greater healthcare resource utilisation and cost.^56^ Conversely, analysis of 7,907 National Surgical Quality Improvement Program (NSQIP) cases by Marquez et al.^57^ found a lower 30-day reoperation rate after delayed-immediate than immediate free-flap reconstruction (9.0% vs. 13.2%; *p <* 0.001), with no significant differences between delayed-immediate and purely delayed reconstruction in surgical complications, length of stay, or operative time. These apparently divergent findings likely reflect differences in study design and observation windows: short-term databases primarily capture morbidity surrounding definitive flap transfer, whereas longitudinal analyses incorporate complications arising during both the expander and flap stages. Overall, the available evidence therefore supports the feasibility of the delayed-immediate pathway but does not establish the bridging interval itself as a low-morbidity phase.

When definitive autologous reconstruction is selected, the timing of flap transfer should be considered separately from the timing of expander-to-implant exchange. In a series of 189 delayed abdominal free-flap reconstructions, Baumann et al.^58^ found that all flap losses occurred in patients reconstructed within 12 months of completing PMRT (overall flap-loss rate 2.6%; *p =* 0.014), and reoperation was more frequent within 12 months than beyond this interval (14.6% vs. 4.7%; *p =* 0.022). Ramaut et al.^59^ similarly describe completion of free-flap reconstruction once irradiated tissues have sufficiently recovered, typically within a 6–12-month interval after PMRT. Although the available evidence does not establish a universal threshold, these data suggest that the timing of definitive autologous reconstruction should be guided by recovery of irradiated tissues and evidence specific to free-flap transfer rather than extrapolated from the approximately 6-month interval commonly considered for expander-to-implant exchange.

Taken together, these data position delayed-immediate reconstruction not as a default intermediate pathway, but as a selective strategy for managing oncological and reconstructive uncertainty, preserving the breast envelope and future options until the definitive reconstructive pathway can be established under more predictable conditions.

## Discussion

The findings of this review suggest that the contemporary role of the tissue expander is defined less by its ability to expand the mastectomy skin envelope than by the flexibility it introduces into the reconstructive pathway.

Fig. 1 illustrates four complementary functions: preservation of the breast envelope, control of volume and tension, flexibility in oncological sequencing, and preservation of future reconstructive options. These functions are particularly valuable when definitive reconstruction cannot or should not be established at mastectomy. Initial filling can be adapted to flap perfusion and tissue tolerance, while subsequent decisions can incorporate final pathology, radiotherapy requirements, tissue evolution, and patient preferences.

**Fig. 1:**
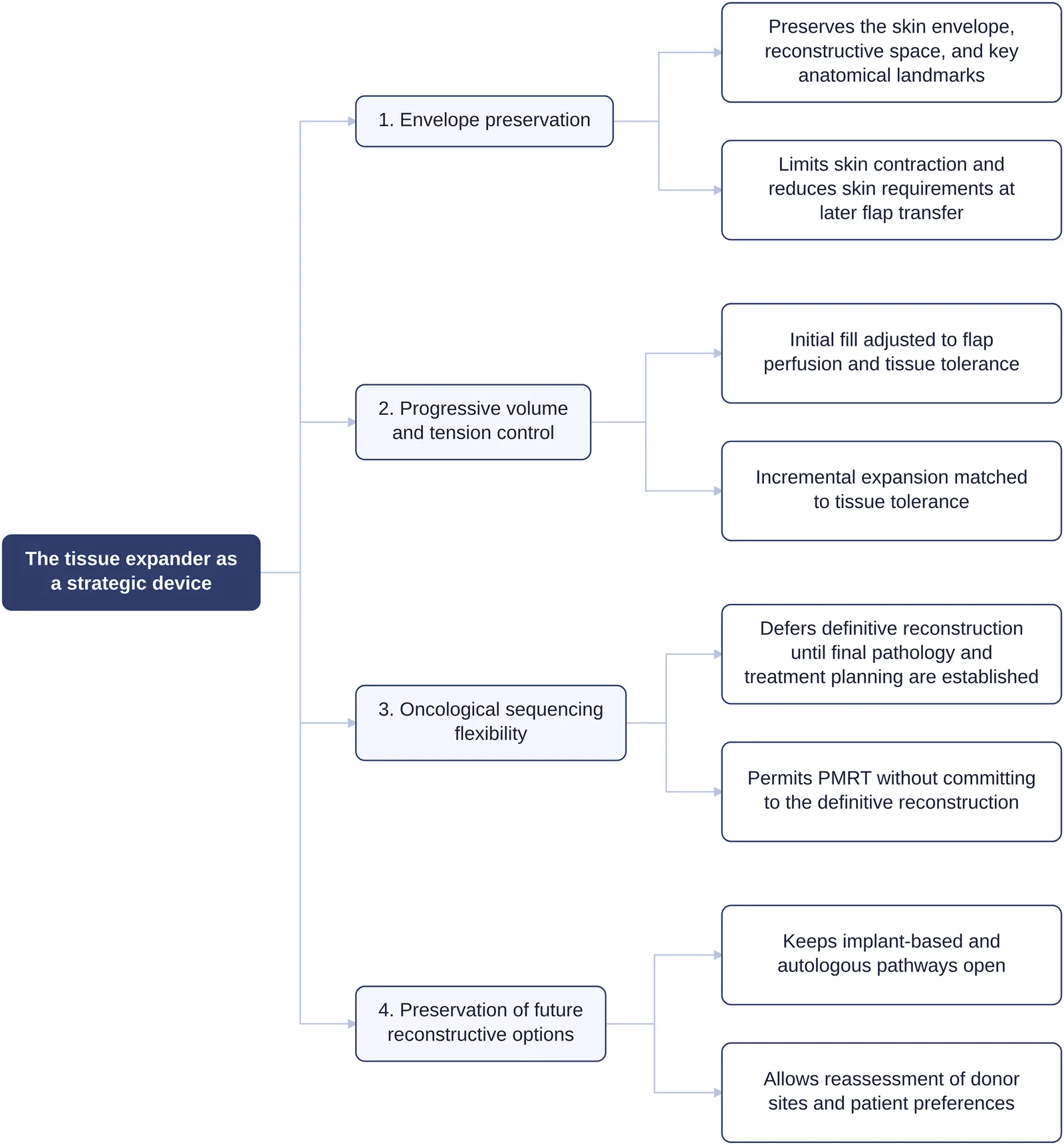
The tissue expander as a strategic device.

This flexibility must be balanced against the additional procedures and device-related morbidity inherent to staged reconstruction. Delayed-immediate data also suggest that the bridging interval is not necessarily a low-morbidity phase. Tissue expander reconstruction should therefore not be regarded as a routinely safer alternative, but as a deliberate strategy when adjustability and preservation of future options provide a clinical advantage.

Fig. 2 translates this concept into a decision framework integrating breast anatomy, anticipated PMRT, comorbidities, and the intended definitive reconstruction. Rather than establishing a hierarchy between DTI, tissue expander, and autologous reconstruction, the algorithm emphasises selective use. DTI is an efficient definitive option when anatomy and patient-related risk factors are favourable and the reconstructive pathway is predictable. Unfavourable anatomy should prompt reconsideration of autologous reconstruction; if an implant-based strategy is pursued, staging may provide greater control of volume and tension.

**Fig. 2:**
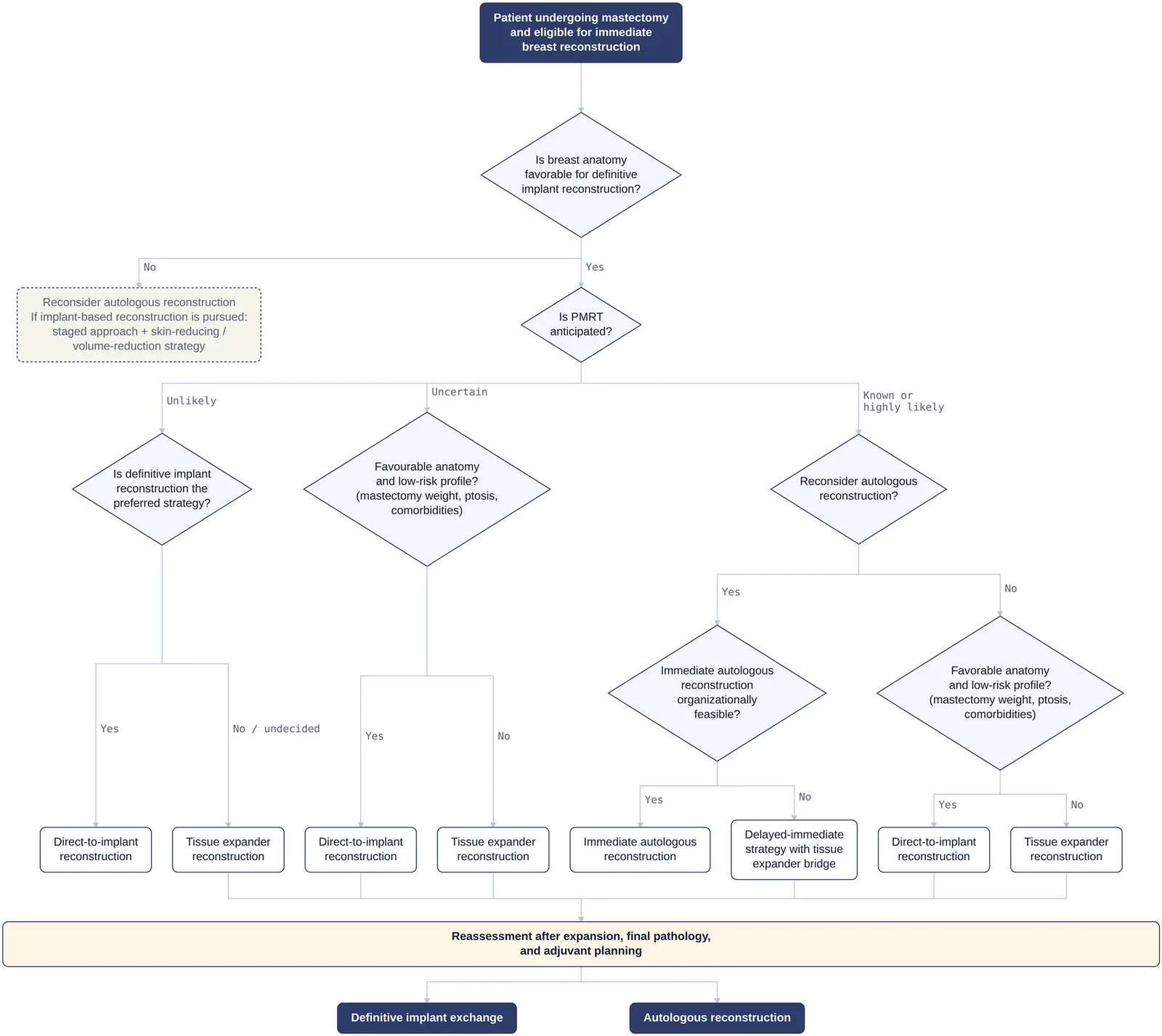
Decision algorithm for immediate breast reconstruction.

PMRT further illustrates the importance of reconstructive flexibility. Its anticipated need should be incorporated into the initial plan without defining a single preferred pathway, and reconstruction should adapt to the oncological timetable rather than delay indicated radiotherapy. When PMRT remains uncertain, breast morphology and patient risk may help determine whether DTI remains reasonable or whether an expander is preferable. When PMRT is known or highly likely, autologous reconstruction should also be reconsidered. Immediate autologous reconstruction may be appropriate when patient and organisational conditions permit, whereas delayed-immediate reconstruction can preserve the breast envelope when flap reconstruction is deferred.

Beyond current reconstructive pathways, emerging oncological sequencing strategies and device innovations may further redefine the role of the tissue expander.

Premastectomy radiotherapy could reduce the need for temporary expander-based sequencing by completing radiotherapy before definitive reconstruction.^60^ Early experience, predominantly involving immediate autologous reconstruction, has been encouraging.^61^ More recently, a phase II trial comparing conventional and hypofractionated premastectomy radiotherapy reported no complete autologous flap losses, although mastectomy-flap necrosis remained clinically relevant and both patients reconstructed with tissue expanders developed infectious complications, with one requiring explantation.^62^ Premastectomy radiotherapy therefore remains promising but investigational, with current evidence supporting its use primarily within carefully selected autologous reconstruction pathways rather than routine expander-based reconstruction.

Technological innovation may also refine the mechanical role of the tissue expander. Dual-chamber devices permit differential filling and more selective control of lower-pole expansion, potentially improving breast shaping while reducing tension around the mastectomy incision. Although preliminary experience is encouraging, current evidence remains insufficient to demonstrate a meaningful clinical advantage over conventional expanders.^63^ Their future relevance will depend on whether improved control of expansion translates into fewer complications and revisions, and ultimately into better aesthetic and patient-reported outcomes.

Overall, the contemporary value of the tissue expander lies less in its ability to expand skin than in its ability to preserve options**.** It maintains the breast envelope and reconstructive space, allows volume and tension to be adjusted, and preserves flexibility while the oncological and reconstructive pathway evolves.

This flexibility comes at the cost of additional procedures and expander-related morbidity, and current evidence does not support routine superiority over DTI or immediate autologous reconstruction. The key issue is therefore not which technique is intrinsically better, but which strategy best matches the patient’s anatomy, risk profile, oncological pathway, and reconstructive objectives.

The tissue expander should thus be regarded neither as a default intermediate step nor as a compromise, but as a deliberately selected tool for managing reconstructive uncertainty. Future studies should therefore ask not whether tissue expander reconstruction is superior, but in which patients its adjustability provides a meaningful clinical benefit.

## Conclusion

Immediate tissue expander breast reconstruction remains relevant not because it is universally safer or superior, but because it preserves flexibility when the reconstructive pathway cannot be definitively established at mastectomy. It allows control of volume and tension, accommodates oncological treatment, and preserves both implant-based and autologous options.

The tissue expander should therefore be viewed as a deliberately selected tool for managing reconstructive uncertainty rather than as a default intermediate step. Future studies should identify the patients in whom this adjustability provides a meaningful clinical benefit.

No fundings.

Ethical Approval was not required.

## Declaration of generative AI and AI-assisted technologies in the manuscript preparation process

During the preparation of this work, the authors used ChatGPT (OpenAI) to assist with English-language translation and editorial refinement, and with the graphic preparation of schematic figures and decision algorithms. All scientific content, interpretation of the literature, and final wording were reviewed and validated by the authors, who take full responsibility for the content of the published article.

## Declaration of competing interest

The authors declare no conflict of interest.
